# Uniting Epidemiology and Experimental Disease Models for Alcohol-Related Pancreatic Disease

**DOI:** 10.35946/arcr.v38.2.03

**Published:** 2017

**Authors:** Veronica Wendy Setiawan, Kristine Monroe, Aurelia Lugea, Dhiraj Yadav, Stephen Pandol

**Affiliations:** Veronica Wendy Setiawan, Ph.D., is an Assistant Professor at the Keck School of Medicine, University of Southern California, Los Angeles, California. Kristine Monroe, Ph.D., is an Associate Professor, at the Keck School of Medicine, University of Southern California, Los Angeles, California. Aurelia Lugea, Ph.D., is an Associate Professor at Cedars-Sinai Medical Center, Los Angeles, California. Dhiraj Yadav, M.D., M.P.H., is a Professor of Medicine in the Division of Gastroenterology & Hepatology, University of Pittsburgh Medical Center, Pittsburgh, Pennsylvania. Stephen J. Pandol, M.D., is Director of Basic and Translational Pancreas Research at Cedars-Sinai Medical Center, Los Angeles, California

**Keywords:** Alcohol-related disease, alcohol-related pancreatic disease, pancreas, pancreatitis, pancreatic cancer, epidemiology, smoking, animal models, experimental disease models

## Abstract

Findings from epidemiologic studies and research with experimental animal models provide insights into alcohol-related disease pathogeneses. Epidemiologic data indicate that heavy drinking and smoking are associated with high rates of pancreatic disease. Less clear is the association between lower levels of drinking and pancreatitis. Intriguingly, a very low percentage of drinkers develop clinical pancreatitis. Experimental models demonstrate that alcohol administration alone does not initiate pancreatitis but does sensitize the pancreas to disease. Understanding the effects of alcohol use on the pancreas may prove beneficial in the prevention of both pancreatitis and pancreatic cancer.

Inflammation of the pancreas, or pancreatitis, can occur suddenly (i.e., acute pancreatitis) or after a long period of damage (i.e., chronic pancreatitis). Chronic pancreatitis is characterized by inflammation that does not improve, and becomes worse over time. Gallstones are a common cause of acute pancreatitis, which is usually resolved with adequate treatments in a few days. Heavy alcohol use over many years is the most common cause of chronic pancreatitis (Yadav and Lowenfels 2013), but cystic fibrosis, tobacco smoking, autoimmune conditions, high levels of calcium or fat in the blood, and certain medications can also cause chronic pancreatitis (National Institute on Diabetes and Digestive and Kidney Diseases 2016). Chronic pancreatitis can lead to diabetes and pancreatic cancer (Yadav and Lowenfels 2013). Since there are no current methods for treating pancreatitis or preventing recurrent episodes of nongallstone-related pancreatitis,understanding the risk factors for this condition is critical to prevention.

Following a review of the epidemiology of both acute and chronic pancreatitis, and pancreatic cancer and the influence of alcohol use and other risk factors, this article examines current experimental models that explore alcohol’s role in pancreatic disease and the cellular mechanisms at work. It focuses on the currently accepted view of alcohol-related pancreatic disease, which holds that alcohol mediates the progression from acute to chronic disease through a number of mechanisms. Following recurrent acute attacks, alcohol may trigger changes leading to chronic pancreatitis and pancreatic cancer. This can happen through alterations in cell signaling pathways; the toxic effects of alcohol’s metabolites on pancreatic cells; oxidative stress; and by promoting activation of pancreatic stellate cells (PSCs), which play an important role in the development of scarring (i.e., fibrosis), inflammation, and tissue damage.

## The Burden of Pancreatic Diseases

Acute pancreatitis is among the most common gastrointestinal causes of inpatient admission to U.S. hospitals. The annual incidence of acute pancreatitis ranges from 13 to 45 per 100,000 people, and chronic pancreatitis from 2 to 14 per 100,000 (Machicado et al. 2016; Yadav and Lowenfels 2013). The incidence of chronic pancreatitis in European countries varies from 1.8 cases per 100,000 people in the Netherlands (Spanier et al. 2013) to 13.4 cases per 100,000 in Finland (Jaakkola and Nordback 1993). A population-based U.S. study noted little change in the incidence of chronic pancreatitis between two time periods (from 3.3 in 1940–1969 to 4.0 per 100,000 in 1977–2006). In Japan, however, a progressive increase in incidence from 5.4 in 1994 to 11.9 in 2007 and 14.0 in 2014 has been noted (Machicado et al. 2016).

Prevalence estimates for chronic pancreatitis are limited to only a few countries (Machicado et al. 2016). Although these rates vary widely, from 13.5 per 100,000 in China to 126 per 100,000 in India, estimates show less variability in the United States, France, Spain, and Japan, ranging from 25 to 50 per 100,000. Similar to incidence, prevalence estimates from Japan increased from 28.5 per 100,000 people in 1994 to 52.4 per 100,000 people in 2014 (Machicado et al. 2016). A 10-year study of patients at 22 hospitals in China also found an increasing prevalence (from 3.08 cases per 100,000 people in 1996 to 13.52 per 100,000 in 2003) (Wang et al. 2009). Although acute pancreatitis affects men and women equally, chronic pancreatitis, especially alcohol-related cases, is more common among men (Yadav and Lowenfels 2013).

Pancreatic cancer accounts for about 3 percent of all cancers in the United States and about 7 percent of cancer deaths (American Cancer Society 2016). Worldwide, the annual incidence rate for pancreatic cancer is about 8 per 100,000 people (Yadav and Lowenfels 2013). Both pancreatitis and pancreatic cancer affect Blacks more than Caucasians, although the reasons for this racial disparity are unclear (Wilcox et al. 2016; Yadav and Lowenfels 2013).

## Progression from Acute to Chronic Pancreatitis

The risk of progression from acute to chronic pancreatitis is higher among alcoholics and smokers, and higher in men than in women. A meta-analysis of 14 studies on this progression concluded that 10 percent of patients with a first episode of acute pancreatitis and 36 percent of patients with recurrent acute pancreatitis develop chronic pancreatitis (Sankaran et al. 2015). Other research found that, following an episode of alcohol-related acute pancreatitis, the risk of progression to chronic pancreatitis was approximately 14 percent with complete abstinence or only occasional drinking, 23 percent with decreased but daily drinking, and 41 percent with drinking at the same level as before the acute episode (Takeyama 2009).

### Morphological Changes in the Pancreas from Acute to Chronic Pancreatitis

Nikkola and colleagues (2014) used imaging technology (secretin-stimulated magnetic resonance cholangiopancreatography) to examine the morphological changes induced by an initial episode of alcoholic pancreatitis. The researchers followed 44 patients after their first episode of alcohol-associated pancreatitis for up to 9 years. They found that whereas a single episode of acute pancreatitis could induce chronic changes, morphological progression (i.e., pancreatic cysts, parenchymal changes, and atrophy) was more frequent in patients with moderate or severe first attacks and in those who had recurrent attacks of pancreatitis.

## Risk Factors for Alcohol-RelatedPancreatic Disease

A meta-analysis of 51 international population-based studies concluded that heavy alcohol use was an impor-tant risk factor for pancreatic disease (Alsamarrai et al. 2014). Overall, the studies demonstrated an estimated 40 percent increased risk of pancreatic disease in heavy drinkers (i.e., those reporting more than 20 drinks per week). The prevalence of pancreatitis is approximately four times higher among people with a history of alcoholism (Yadav et al. 2007). Historically, an estimated 60 to 90 percent of chronic pancreatitis cases were attributed to alcohol use (Coté et al. 2011). However, more recent research suggests a lower prevalence of heavy drinking among chronic pancreatitis patients than previously estimated (Frulloni et al. 2009; Yadav et al. 2009). One recent study estimating the prevalence of alcohol-related pancreatitis used data from 539 patients and 695 unaffected study participants enrolled in a study of pancreatic disease at U.S. treatment centers (Coté et al. 2011). An estimated 44.5 percent of chronic pancreatitis cases were classified as alcohol related, based on physician assessment. The authors acknowledge that the lower-than-expected rate of alcohol-related disease may be due to the specialized nature of the treatment centers, the fact that alcohol users may be less likely to seek care, or because physicians who attribute a patient’s disease to alcohol use would be less likely to refer them to a specialist’s care. In Japan, a questionnaire to assess alcohol use among patients with alcoholic pancreatitis found that women developed pancreatitis at a younger age, with shorter duration of alcohol use, and after smaller cumulative amounts of alcohol consumption compared with male patients (Masamune et al. 2013). In this study, continued drinking led to the recurrence of pancreatitis.

Some studies have suggested a threshold of alcohol use above which there is an increased risk for pancreatitis. Yadav and colleagues (2009) found the threshold to be 5 drinks per day for chronic pancreatitis. The relationship between lower levels of alcohol consumption and pancreas disorders is less well defined. In one recent meta-analysis of seven published studies, researchers noted a dose-dependent relationship between alcohol use and chronic pancreatitis in both sexes and for acute pancreatitis among men (Samokhvalov et al. 2015). Interestingly, a J-shaped relationship for the association with acute pancreatitis was noted among women, with a protective effect at less than 40 grams of ethanol per day (2 to 3 drinks) (Samokhvalov et al. 2015). Another recent study across a large diverse population not included in the meta-analysis observed a protective effect of moderate drinking on recurrent acute or chronic pancreatitis in men, and for all pancreatitis in women (Setiawan et al. 2016). Suggested explanations for this observation are a decreased risk of gallstone formation with moderate drinking, characteristics of the study population (older cohort), difficulty in assessing accurate exposure information, and possible contamination of the control group with former drinkers (Yadav 2016). Biological plausibility for how moderate drinking may have a protective effect is discussed later in this review. Data on the role of type and pattern of alcohol consumption and risk of pancreatitis are too limited to make definitive conclusions.

For pancreatic cancer, results from meta-analyses estimate a 20-percent increased risk from consuming 3 drinks per day (Maisonneuve and Lowenfels 2015; Tramacere et al. 2010). Another meta-analysis of individual participant data for more than 800,000 people found 22 percent increased risk of pancreatic cancer among people who consumed more than 3 drinks per day, although the association was only significant in women (Genkinger et al. 2009). A meta-analysis of alcohol’s impact on risk for 23 types of cancer that included 572 studies found that heavy drinkers had a significantly higher risk of pancreatic cancer (relative risk of 1.19) compared with nondrinkers and occasional drinkers (Bagnardi et al. 2015).

## Alcohol and Smoking Interactions

Cigarette smoking and heavy alcohol use, commonly co-occurring behaviors, increase risk for pancreatitis and pancreatic cancer (Yadav and Whitcomb 2010). A study of 108 smokers with alcohol-related chronic pancreatitis examined disease outcomes in relation to tobacco dose. The researchers concluded that smoking accelerates the course of pancreatic disease in a dose-dependent fashion, separate from the level of alcohol consumption (Rebours et al. 2012). A meta-analysis of 12 studies reported that while smoking increases the risk of chronic pancreatitis independently from alcohol, the effects of smoking are stronger for alcohol-related pancreatitis (Andriulli et al. 2010). In a recent study, Setiawan and colleagues (2016) found that smoking was significantly associated with nongallstone acute and chronic pancreatitis. The risk associated with current smoking was highest among men who consumed more than 4 drinks per day. For pancreatic cancer, among current smokers, heavy alcohol consumption was associated with a significantly increased pancreatic cancer risk. Risk was increased insignificantly among light and moderate drinkers who were smokers (Rahman et al. 2015).

Research comparing pancreatic duct-cell function in current and former smokers with never-smokers found that smoking was an independent predictor of cell dysfunction, after controlling for age, gender, and alcohol intake. The study also found no interaction between smoking status and alcohol consumption in predicting duct-cell dysfunction (Kadiyala et al. 2013).

## Alcohol and Genetic Interactions

Although alcohol abuse and smoking are major environmental risk factors for pancreatic disease, only a small percentage of drinkers and smokers develop pancreatic disease (Yadav and Lowenfels 2013). This has led to a search for a role of genetic differences that could explain the susceptibility of some individuals to the effects of alcohol on the pancreas. Whitcomb and colleagues (2012) identified an association between genetic variants of Claudin-2 (*CLDN2*) and the risk of alcoholic pancreatitis. *CLDN2* is an X-linked gene involved in tight junction permeability and is expressed by pancreatic acinar cells. Alterations in the function of tight junctions in the pancreas or possibly in the intestinal epithelium could inappropriately expose the pancreas to toxins that could interact with the direct effects of alcohol in the pancreas. A recent study (Koziel et al. 2015) concluded that genetic mutations in SPINK1, a protein that inhibits activation of trypsinogens within the pancreas, may predispose individuals to severe acute pancreatitis, especially in patients that abuse alcohol.

As described in these epidemiologic studies (Yadav and Lowenfels 2013), pancreatic disease appears to be triggered by repeated acute attacks in combination with heavy alcohol use and other factors such as smoking and genetic factors.

## Molecular Mechanisms of Alcohol-Related Acute and Chronic Pancreatitis

The general concepts that have been followed in developing animal models for alcohol research are based on observations originally described by Comfort and colleagues (1946). They found histological changes consistent with acute pancreatitis in patients with chronic pancreatitis. When followed longitudinally, these patients had greater amounts of necrosis indicative of acute pancreatitis early in the disease course and fibrosis in later stages, suggesting that chronic pancreatitis developed from repeated attacks of acute pancreatitis.

Studies using animal models of pancreatitis have supported the idea that alcohol-related exocrine pancreatic disease is induced by the combination of ethanol and other factors. For example, cholecystokinin (CCK) analogues cause pancreatitis in rodents in the absence of alcohol treatments only at doses much greater than those needed to activate known physiologic responses such as pancreatic enzyme secretion and gallbladder contraction (Lam et al. 2007). However, in ethanol-fed animals, CCK causes acute pancreatitis when given at more physiologic doses (Pandol et al. 1999). In other examples, ethanol feeding exacerbates pancreatitis due to hyperlipidemia and pancreatic-duct obstruction (Grauvogel et al. 2010). Ethanol-feeding models have also been used to show that alcohol impedes recovery from acute pancreatitis, resulting in promotion of chronic-pancreatitis features of chronic inflammation and fibrosis (Gukovsky et al. 2008).

Other animal models are based on previous observation of the increased susceptibility of people with compromised immunity (a common consequence of alcohol abuse) to viral pancreatitis. Using a mouse model, Jerrells and colleagues (2007) found that ethanol consumption alone does not produce pancreatic damage but causes viral pancreatitis to be more severe and prolonged. Similarly, others have shown that alcohol feeding and lipopolysaccharide (LPS) administration, to mimic the effects of alcohol on increased circulating LPS in humans, promotes pathologic features of chronic pancreatitis (Fortunato et al. 2006; Nakayama et al. 2014; Vonlaufen et al. 2007, 2011). Importantly, Vonlaufen and colleagues (2011) showed in the LPS-alcohol model that alcohol withdrawal causes regression of the features of chronic pancreatitis, indicating the importance of alcohol in promoting disease progression as originally described in humans (Comfort et al. 1946).

To emphasize, alcohol feeding alone had minimal pathologic effects in these models. Furthermore, the initiating agents for causing pancreatitis (i.e., CCK, LPS, duct obstruction, or viral infection) at the doses used in the corresponding models have minimal effects on pancreatitis responses in the absence of alcohol treatments.

## Role of Pancreatic Acinar Cells and Ductal Cells

Research into the molecular mechanisms of alcohol-related pancreatitis has largely focused on the pancreatic acinar cell, the component of the pancreas devoted to synthesis, storage, and secretion of digestive enzymes. These studies suggest that alcohol does not directly damage acinar cells but may make cells susceptible to other factors that trigger cell damage. For example, in vitro and in vivo studies that focus on the effects of CCK on the transcription factor NF-κB, an intracellular signaling pathway involved in the inflammatory response of pancreatitis, show that alcohol treatments augment CCK-induced NF-κB activation (Pandol et al. 1999). Another study suggested that alcohol activates a specific isoform of the signaling molecule known as protein kinase C (i.e., protein kinase C epsilon, PKCɛ), which, in turn, is involved in NF-κB activation and the initiation of pancreatitis (Satoh et al. 2006). Further research using experimental models of acute pancreatitis examined the mechanisms through which PKCɛ regulates cell death. The researchers found that PKCɛ knockout mice (in which PKCɛ is genetically deleted) had decreased inflammation and necrosis and less severe acute pancreatitis in response to high doses of CCK analogues (Liu et al. 2014). In addition, alcohol has been found to promote secretion of digestive enzymes from the basolateral aspect of the acinar cell via mechanisms involving protein kinase C (Cosen-Binker 2007). Basolateral enzyme secretion would inject the digestive enzymes into the tissue of the pancreas where they can cause injury to the pancreas and pancreatitis.

More recently, studies have turned to determining effects of alcohol on the pancreatic duct cell, which is important for producing fluid secretion and carrying digestive enzymes secreted by the acinar cell into the gut lumen, where they are needed for meal digestion. These studies show that excessive alcohol drinking can cause inhibition of the function of the same transporter that is inhibited by mutation in cystic fibrosis (Maléth et al. 2015). These findings, and the fact that the acinar cells and duct cells must both perform their functions in a coordinated fashion to prevent disease (Hegyi et al. 2011), suggest that alcohol can promote pancreatitis via its actions on one or both of the key cellular components of the pancreas.

## Role of Pancreatic Stellate Cells

Alcohol-related pancreatitis has been linked to the activation of pancreatic stellate cells (PaSC) (Apte et al. 1999, 2000; Vonlaufen et al. 2007, 2011). PaSC are normal resident cells in the exocrine pancreas. They are present in the periacinar space and have long cytoplasmic processes that surround the acinar structures and ducts of the exocrine pancreas (Omary et al. 2007).

In their normal state, often referred to as “quiescent,” PaSC provide basement membrane and organization of the pancreatic epithelium. However, in pathologic states such as alcohol-induced pancreatitis, PaSC participate in disease pathogenesis after transforming into an “activated” state (also known as a “myofibroblastic” state) (Omary et al. 2007). These cells target an injured area and play a role in tissue repair (Apte et al. 1999). However, when they develop into a sustained activated state inappropriately, PaSC play a major role in alcohol-related pancreatitis. They mediate both the fibrosis and chronic inflammatory response of chronic alcoholic pancreatitis as well as pancreatic cancer (Apte et al. 2013). Regarding chronic pancreatitis, research suggests that this activation is mediated by alcohol, its toxic metabolite (i.e., acetaldehyde), or oxidative stress. Researchers have sought to identify the intracellular signaling pathways mediating PaSC responses. The goal of such research would be to develop strategies to target specific signaling molecules and interrupt PaSC activation, inhibiting abnormal fibrogenesis.

Recent studies suggest that the mitogen-activated protein kinase (MAPK) pathway, a major intracellular signaling pathway, plays a role in regulating the effects of alcohol and its metabolite acetaldehyde on PaSC (Apte et al. 2007). In addition, alcohol has been shown to activate the membrane-bound enzyme complex nicotinamide adenine dinucleotide phosphate (NADPH) oxidase system, contributing to PaSC proliferation (Hu et al. 2007).

To address the epidemiologic observations of combined effects of alcohol and smoking, Lee and colleagues (2015) showed that cigarette smoking extract as well as nicotine and one of its major metabolites caused activation of PaSC. This activation was mediated via nicotinic acetylcholine receptors they found on the PaSC, and alcohol added to the effects of the smoking molecules.

The following sections summarize other potential co-factors that might trigger alcohol-related pancreatitis, including the participation of ethanol metabolites in alcohol-induced pancreas pathology.

## Ethanol Metabolism in the Exocrine Pancreas

Metabolism of ethanol by the exocrine pancreas occurs by both oxidative and nonoxidative routes (Gukovskaya et al. 2002; Haber et al. 2004). The oxidative pathway is the predominant pathway for ethanol elimination in the body, occurring mostly in the liver. In the oxidative pathway, ethanol is converted to acetaldehyde by alcohol dehydrogenases (ADH), and then acetaldehyde is converted to acetate by mitochondrial aldehyde dehydrogenases (ALDH). Both enzymes are functional and present in the exocrine pancreas. The nonoxidative route of ethanol metabolism involves covalent coupling of ethanol with fatty acids to yield lipophilic fatty acid ethyl esters (FAEEs). This pathway provides the transient storage of ethanol while it awaits oxidative metabolism for removal from the body. The importance of the nonoxidative pathway comes from observations that humans dying from alcohol intoxication have high levels of FAEEs in the pancreas (Laposata and Lange 1986) and the finding that the FAEEs are formed using the enzyme carboxylester lipase, a highly expressed digestive enzyme made in the pancreas and secreted during lipid digestion (Huang et al. 2014).

There has been increasing evidence that the nonoxidative pathway plays an important role in alcohol pathogenesis in the acinar cell. For example, FAEEs were found to cause necrosis in pancreatic acinar cells by inducing sustained increases in free concentrations of Ca^2+^ in the cytoplasm from released intracellular stores, leading to toxicity of mitochondria and failure to produce ATP (Criddle et al. 2004, 2006). In addition, FAEE administration to experimental animals causes pancreas pathology (Lugea et al. 2003). Moreover, studies using pharmacologic and genetic inhibition of ADH caused pancreatitis responses in animal models, while pharmacologic inhibition of carboxylester lipase inhibited pancreatitis responses (Huang et al. 2014; Kaphalia et al. 2010).

Several genetic polymorphisms in the enzymes metabolizing ethanol have been described in humans in the last decade. A recent review by Aghdassi and colleagues (2015) summarizes these polymorphisms and their potential for conferring high susceptibility to alcohol-related pancreatic disorders. The most common polymorphism, an inactive ALDH2 gene, affects 40 to 50 percent of East Asians who exhibit high levels of acetaldehyde in blood after alcohol consumption, and higher susceptibility to acetaldehyde toxicity and certain forms of cancer (Chao et al. 2000; Yokoyama et al. 2010).

However, studies on the relevance of specific genetic polymorphisms of ethanol-metabolizing enzymes on pancreatic disorders have been limited, and the resulting data equivocal. Future studies will help to clarify whether these polymorphisms alone or in combination alter the susceptibility to alcohol-related chronic pancreatitis and pancreatic cancer.

## Alcohol and the Cholinergic System

The neurotransmitter acetylcholine may play a role in alcohol-induced pancreatic damage. Lugea and colleagues (2010) found that atropine dramatically reduced cerulein-induced pancreatitis in alcohol-fed rats, indicating that alcohol-ensitizing effects are mediated at least in part through activation of cholinergic pathways. This effect is independent of the effects of smoking on nicotinic receptors present on the PaSC, described below.

## Alcohol and Mitochondrial Dysfunction

Mitochondrial membrane permeabilization (MMP) triggers mitochondrial dysfunction and cell death and leads to tissue damage. The mitochondrial permeability transition pore (MPTP) plays a critical role in MMP. Research with pancreatic cells from mice found that oxidative metabolism of ethanol sensitizes mitochondria to activate MPTP, making them more sensitive to the toxicity by low concentrations of Ca^2+^ in the cell. This leads to mitochondrial failure and ATP depletion, making the pancreas susceptible to pancreatitis (Huang et al. 2014; Shalbueva et al. 2013).

## Alcohol, Autophagy, and Lysosomes

Autophagy is a natural and regulated process for the cell to disassemble unnecessary or dysfunctional components. This disassembly allows for an orderly recycling of cellular components. The process of autophagy involves isolating targeted cellular constituents within a double-membrane vesicle known as the autophagosome. The autophagosome eventually fuses with the cell’s lysosomes to form a compartment where lysosomal enzymes carry out the disassembly. Recent studies have shown the importance of normal autophagy and lysosomal function in the mechanism of pancreatitis (Gukovskaya et al. 2016). That is, animal models created with genetic inhibition of key autophagic mediators (i.e., autophagy protein 5, Atg5, or Atg7) or the glycoprotein required for lysosomal integrity (i.e., lysosomal-associated membrane protein-2, LAMP2) lack normal autophagic processing, resulting in inappropriate processing of digestive enzymes in the acinar cells and spontaneous pancreatitis. Further, in nonalcoholic models of pancreatitis, findings of disordered fusion and function of the lysosomal-autophagic system have been described (Gukovskaya et al. 2016).

Several studies have demonstrated the effects of alcohol on lysosomal and autophagy function. For example, Wilson and colleagues (Haber et al. 1993; Wilson et al. 1990, 1992) demonstrated that an alcohol diet or treatment of isolated lysosomes with FAEEs or cholesteryl esters caused lysosomal fragility and leakage of lysosomal enzymes into the acinar cell cytosol. Furthermore, more recent studies show that alcohol feeding and LPS treatment decrease the expression of LAMP2 in the pancreas of animals (Fortunato et al. 2009; Mareninova et al. 2015). In sum, these studies show that alcohol feeding, FAEE, and LPS cause lysosomal and autophagy dysfunction, which may result in pancreatitis responses.

### Dietary Factors

### Thiamine Deficiency

Thiamine (vitamin B1) is essential for pancreatic acinar-cell function. Cells obtain thiamine from their surroundings and enzymatically convert it into thiamine pyrophosphate (TPP), which is transported to mitochondria by the mitochondrial TPP transporter (MTPPT). Srinivasan and colleagues (2015) found that, in mice, chronic alcohol exposure significantly inhibited TPP uptake, which was associated with decreased expression of MTPPT protein and activity of the gene for MTPPT in pancreatic acinar cells. The authors suggest that this effect of alcohol could have a negative effect on physiologic function of the mitochondria in the acinar cell and make them susceptible to pathologic responses with stress.

### Folate Deficiency

Dietary folate is critical for pancreatic health. A study in rats receiving a chronic alcohol diet found a significant decrease in folate uptake by isolated pancreatic cells compared with rats not receiving alcohol. The alcohol-fed rats also had decreased activity in both of the major folate uptake systems (i.e., reduced folate carrier and proton-coupled folate transporter) (Said et al. 2010).

### Fiber

A population-based prospective analysis of dietary factors for pancreatitis in the United States found that the majority of dietary factors were mainly associated with the risk of gallstone-related pancreatitis, with the notable exception of dietary fiber (Setiawan et al. 2017). The investigators found dietary fiber to be inversely associated with both gallstone- and nongallstone-related acute pancreatitis but not suspected chronic pancreatitis. Fiber has been associated with changes in gut microbiota, improvements in gut epithelial tightness, and prevention of endotoxin transit into the system (Blaut 2015; Ghanim et al. 2009). Importantly, experimental animal models of pancreatitis show that endotoxin can promote the development and severity of pancreatitis (Fortunato et al. 2006; Vonlaufen et al. 2007). Insoluble fiber may also have a protective effect by reducing the development of gallstones (Tsai et al. 2004), a major cause of acute pancreatitis. Dietary fiber has also been associated with reduced pancreatic cancer risk (Wang et al. 2015).

### Vitamin D

Vitamin D deficiency is associated with several disorders. However, epidemiological data linking vitamin D deficiency to an increased risk for alcoholic and nonalcoholic chronic pancreatitis or pancreatic cancer are scarce and inconsistent (Hoogenboom et al. 2016; Waterhouse et al. 2016).

In experimental settings, a recent study found that a vitamin D agonist decreases features of chronic pancreatitis, including fibrosis and inflammation (Sherman et al. 2014), supporting the participation of vitamin D signaling in the development of pancreas scarring. Further research should clarify the clinical relevance of the experimental data.

## Alcohol-Induced Adaptive Systems and Pancreatitis

Despite the increased risk for pancreatic damage among heavy drinkers, the incidence of clinical pancreatitis in heavy drinkers is low (~5 percent) (Yadav et al. 2007). One potential explanation for the low rate of pancreatitis among heavy drinkers is that alcohol induces adaptive systems that serve to protect the pancreas from the damaging effects of alcohol. This theory holds that disease progresses when the damaging effects are stronger than the protective effects, or when the protective systems are impaired. Thus, the combination of alcohol use and another risk factor could represent an overwhelming burden and therefore lead to disease progression.

Research using animal models has examined the role of a cellular stress response (i.e., the unfolded protein response, UPR) as an adaptive response to heavy alcohol use that may protect the pancreas from alcohol’s damaging effects (Lugea et al. 2015; Pandol et al. 2011). The UPR is critical for efficient functioning of the endoplasmic reticulum (ER) in the pancreatic acinar cell, because the ER provides for the synthesis of cellular components necessary for transporting digestive enzymes manufactured in ER to zymogen granules for storage and for secretion.

Lugea and colleagues (2011) examined this protective effect in mice with and without the gene for the X-box binding protein 1 (XBP1), a transcription factor that promotes synthesis of cellular components for protein transport and secretion. XBP1 is a key regulator of the adaptive UPR in the pancreas. The researchers found that ethanol feeding in control mice causes a marked increase in the activated form of XBP1 associated with minor pancreatic damage. But in mice with an inability to increase activated XBP1, ethanol feeding results in pancreatic damage. This protective response stimulated by alcohol may be one reason why so few alcoholics develop pancreatic disease. The results of the experiments suggest that enhancing the protective responses may provide opportunities for prevention and treatment of pancreatic diseases.

## Molecular Mechanisms of Alcohol-Related Pancreatic Cancer

Most genetically engineered mouse models of pancreatic cancer are based on genetic mutations in the *Kras* gene. Mice expressing mutant *Kras* develop early and advanced forms of the most common pancreatic cancers in humans. However, *Kras* mutations alone are not sufficient to induce progression to the invasive stage of pancreatic cancer. Rather, different transgenes have been used to create models that progress to invasive cancer. For example, one common model based on *Kras* mutations is the PDX1-Cre;LSL-Kras^G12D^ model. Xu and colleagues (2015) reported using this model in mice exposed to alcohol and given injections of cerulein. The mice developed fibrosis and had an increased level of cancerous lesions. The authors concluded that alcohol independently increased pancreatic-cancer risk associated with fibrosis. Another animal model induces pancreatic cancer through the implantation of dimethylbenzanthracene (DMBA) in the pancreas. Research using this method in mice resulted in the development of both precursor lesions and invasive tumors. There was a higher relative frequency of tumors in mice receiving alcohol compared with the control group (Wendt et al. 2007).

The precise molecular mechanisms by which alcohol use may promote the development and/or progression of pancreatic cancer are not well defined. Although not evaluated in experimental models of pancreatic cancer, the oxidative ethanol metabolite acetaldehyde can act as a carcinogen by forming DNA adducts (Yu et al. 2010). In addition, alcohol might favor cancer development by causing oxidative stress and lipid peroxidation. Alcohol abuse may also accelerate tumor progression by promoting pancreatic inflammation. In this respect, studies using mouse models of pancreatic cancer demonstrated that recurrent pancreatic inflammation is required for the transformation of premalignant lesions into pancreatic cancer (Guerra et al. 2007), and epidemiologic studies indicate that chronic pancreatitis is a major risk factor for pancreatic cancer in humans (Duell et al. 2012). Finally, recent studies have shown that alcohol use may induce epigenetic changes, mainly histone acetylation and DNA methylation, which affect expression of many genes. However, the full involvement of epigenetic mechanisms in alcohol-related chronic pancreatitis or pancreatic cancer has yet to be investigated.

## Conclusions

The combination of epidemiologic and experimental animal-model observations continues to reveal insights into both disease pathogenesis and potential adaptive protective mechanisms of alcohol use. The relationship between heavy alcohol consumption and acute and chronic pancreatitis is well established (Yadav 2016). The highest rates of nongallstone-related pancreatitis are observed in those who drink the greatest amount of alcohol. A recent epidemiological observation of a potential protective effect of moderate alcohol use should be considered preliminary, encourage further research to confirm and determine generalizability of these findings, and elucidate the potential mechanism. Further, smoking is associated with significant risk for non-gallstone-related pancreatitis and may add to the risk of pancreatitis with heavy drinking. A very low percentage of drinkers develop pancreatitis. Experimental models demonstrate that alcohol administration alone may not initiate pancreatitis, but it sensitizes the pancreas to pancreatitis by other insults.

Work in these models also reveals that the pancreas adapts to alcohol administration using the endoplasmic reticulum-based UPR to prevent injury. There is increasing interest in the role of carboxyester lipase, a pancreatic digestive enzyme, in forming fatty acid ethyl esters, which exert toxic effects through sustained increases in intracellular Ca^2+^ concentrations. These in turn cause mitochondrial failure and decreased ATP production necessary to prevent cellular necrosis. The effects of alcohol use on pancreatic-cancer risk are largely through its promotion of repeated episodes of acute inflammatory pancreatitis and chronic pancreatitis. Understanding and preventing the injurious effects of alcohol use on the pancreas resulting in pancreatitis will likely also have a large benefit on prevention of pancreatic cancer. The figure presents a summary of epidemiologic and mechanistic findings in an attempt to provide an impetus for further developments in the field.

## Figures and Tables

**Figure:**
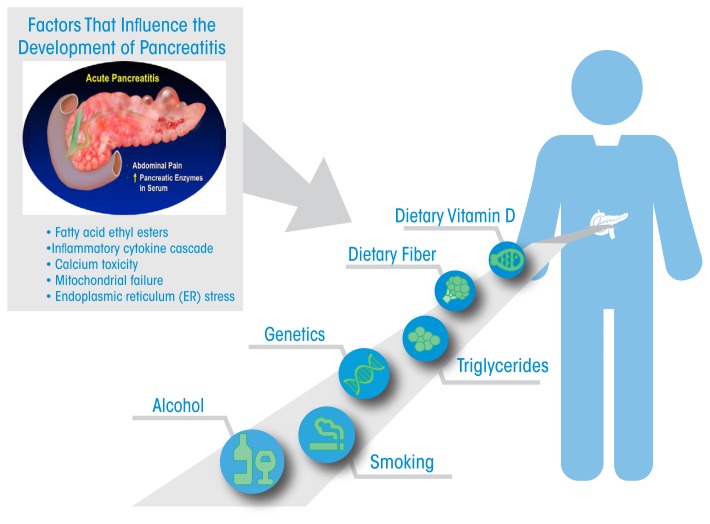
The figure emphasizes the association of alcohol abuse, smoking, high triglycerides, and specific genetic mutations in promoting pancreatic disease. Dietary fiber and vitamin D are associated with protection from pancreatitis. The insert in the upper-left aspect of the figure shows the factors in the pancreatic tissue that are involved in the mechanisms of pancreatitis development.
